# Efficacy and safety of immunosuppressive therapy versus cyclosporine combined with avatrombopag in older adults with severe aplastic anemia: a multicenter prospective study

**DOI:** 10.1038/s41408-025-01328-3

**Published:** 2025-07-05

**Authors:** Leyu Wang, Lei Ye, Xin Zhao, Miao Chen, Fengkui Zhang, Bing Han

**Affiliations:** 1 Department of Hematology, Peking Union Medical College Hospital, Chinese Academy of Medical Science and Peking Union Medical College, Beijing, China; 2 State Key Laboratory of Experimental Hematology, National Clinical Research Center for Blood Diseases, Haihe Laboratory of Cell Ecosystem, Institute of Hematology & Blood Diseases Hospital, Chinese Academy of Medical Sciences & Peking Union Medical College, Tianjin, China

**Keywords:** Clinical trial design, Immunotherapy

## Abstract

This trial compared antithymocyte globulin (ATG) + cyclosporine A (CsA) + avatrombopag (AVA) and CsA + AVA in older adults with severe aplastic anemia (SAA). The patients were randomized to receive either ATG + CsA + AVA or CsA + AVA. Of 84 included patients, 42 were treated with ATG + CsA + AVA and 42 with CsA + AVA. With a median follow-up of 13 (0.3–17) months, the objective response rates (ORRs) at 3, 6, and 12 months and the end of follow-up were 53.7%, 65.9%, 80.6%, and 71.4% in the ATG + CsA + AVA group and 61.9%, 73.2%, 77.4%, and 64.3% in the CsA + AVA group, respectively (P > 0.05 at any time point). Three-month ORR was an independent predictor of 6-month complete response rates (P = 0.019). Patients in the ATG + CsA + AVA group showed a higher incidence of adverse events than those in the CsA + AVA group (64.3% vs. 35.7%, P = 0.009). The rates of relapse (P = 0.667), mortality (P = 1.000) and clonal evolution (P = 1.000) were comparable between the groups. The combination of CsA + AVA achieved comparable efficacy with superior safety compared to the combination of ATG + CsA + AVA in older adults newly diagnosed with SAA.

## Introduction

Aplastic anemia (AA) is a bone marrow failure syndrome characterized by potentially life-threatening bone marrow hypoplasia and cytopenia [1]. For patients with severe aplastic anemia (SAA) who are ineligible for hematopoietic stem cell transplantation (HSCT), intensive immunosuppressive therapy (IST) with antithymocyte globulin (ATG) and cyclosporine A (CsA) is the preferred treatment option [2]. The response rate of patients with SAA to ATG + CsA is 60–80% [3–5], however, approximately 10–30% of the patients treated with IST relapsed during the follow-up period [5, 6].

Thrombopoietin (TPO) is an endogenous factor that regulates megakaryocyte proliferation and platelet (PLT) production [7]. TPO receptor agonists (TPO-RAs) bind to the TPO receptor, inducing a conformational change. This activates the Janus Kinase 2/Signal Transducer and Activator of Transcription (JAK2/STAT) signaling pathway, which in turn promotes the proliferation of megakaryocyte progenitor cells and the production of PLTs [8]. The current indications for TPO-RA include the treatment of chronic immune thrombocytopenia (ITP) in patients with inadequate response to prior therapy using options such as avatrombopag (AVA), eltrombopag (EPAG), herombopag, and romiplostim; thrombocytopenia with chronic liver disease (CLD) in adult patients who are scheduled for surgery using AVA and lusutrombopag; SAA using EPAG, hetrombopag, and romiplostim; and interferon-based hepatitis C using EPAG [9–11]. Combining EPAG to standard ATG + CsA regimens can improve hematologic responses in patients with newly diagnosed SAA [12, 13]. However, ATG is relatively expensive, requires hospitalization for administration, and is associated with a relatively high incidence of adverse reactions, which limit its use, particularly in older adults with SAA. Our previous retrospective study has emphasized that the overall response rate (ORR) of patients who received CSA + EPAG was not inferior to patients with newly diagnosed SAA who received ATG + CsA + EPAG [14]. On the other hand, EPAG is also associated with a number of adverse reactions, particularly liver function impairment [12, 15] which also raises concerns for its use in older adults.

AVA is a novel TPO-RA approved by the US Food and Drug Administration for treating ITP in patients with inadequate response to prior therapy, as well as in patients with CLD [16, 17]. Several clinical trials have shown that, compared to other TPO-RAs, AVA is superior in treating certain thrombocytopenic diseases, such as primary ITP, AA, and chemotherapy-induced thrombocytopenia [18–22]. Our previous studies have shown that AVA can achieve an efficacy comparable to that of EPAG, and produces a faster PLT response in patients with refractory or relapsed AA [20]. Additionally, AVA has the advantage of fewer side effects, making it more convenient for older patients [23, 24]. To date, no prospective studies have directly compared the therapeutic efficacy of ATG + CsA + AVA and CsA + AVA regimens in older adults. Therefore, we designed a randomized clinical trial to compare the response, safety, and clonal evolution in patients treated with these regimens, and further analyzed factors that potentially influence the response rates.

## Materials/subjects and methods

This multicenter, prospective, randomized controlled study was approved by the ethics committee of Peking Union Medical College Hospital (PUMCH) and registered at clinicaltrials.gov (NCT05996393). All eligible patients were fully informed about the study design, and provided written informed consents for their participation. Withdrawal from the trial was permitted at any stage, at the patient’s request.

### Patient characteristics

Patients diagnosed with SAA in Peking Union Medical College Hospital (PUMCH) and Institute of Hematology and Blood Diseases Hospital, Chinese Academy of Medical Sciences, Peking Union Medical College from September 2023 to August 2024 were prospectively enrolled according to the following criteria: (1) age ≥ 60 years; (2) diagnosis of SAA or very severe aplastic anemia (VSAA); (3) no prior treatment with cyclosporine, tacrolimus or hormones; (4) no prior treatment with TPO-RAs (such as thrombopoietin, EPAG, and herombopag); (5) documented patient consent.

The exclusion criteria were as follows: (1) presence of other potential causes of pancytopenia; (2) evidence of other hematological bone marrow disorders, such as acute myeloid leukemia (AML) or myelodysplastic syndromes (MDS); (3) paroxysmal nocturnal hemoglobinuria (PNH) clone ≥50%; (4) history of hematopoietic stem cell transplant before enrollment; (5) presence of bleeding or infection that could not be controlled with standardized treatments; (6) presence of active infection, hepatitis, cirrhosis or portal hypertension; (7) presence of malignant tumors; (8) history of thromboembolic events, such as acute coronary syndrome or stroke treated with anticoagulants; (9) patients with severe renal insufficiency having serum creatinine levels >2 times the upper limit of normal (ULN); (10) albumin transaminase level >2.5 times the ULN and total bilirubin level >2.5 times the ULN ; (11) participation in other clinical trials within the last 3 months and (12) pregnant or lactating women. The diagnosis and severity of SAA and VSAA were classified based on previously described criteria [25] After signing the consent forms, the patients were assigned into either the CSA + ATG + AVA group or the CsA + AVA group according to computer-generated random numbers.

### Intervention

Regarding the ATG + CsA + AVA group, CsA was administered at a dosage of 3–5 mg/kg/day, and the trough plasma blood concentration was maintained at 100–200 ng/ml for at least six months, with a gradual reduction of the dosage after three months for patients showing optimal efficacy. Rabbit ATG was administered at a dosage of 3–5 mg/kg/day for five days and AVA was administered at a daily oral dose of 60 mg, with dose adjustments based on the PLT count. Regarding the CsA + AVA group, CsA and AVA were administered at a dose similar to that administered in the ATG + CsA + AVA group. The patient was eligible to receive a transfusion if the hemoglobin (HGB) level was < 60 g/L and/or the PLT count was <20×10^9^/L, or whenever required. Granulocyte colony-stimulating factor was administered when the absolute neutrophil count (ANC) was <1.0×10^9^/L.

### Endpoints

The primary endpoint was the hematological response at 6 months, which was represented by the ORR, including complete (CR) and partial response (PR). CR was defined as HGB > 120 g/L for males (110 g/L for females), PLT count >100×10^9^/L, and ANC > 1.5×10^9^/L [25]. PR was defined as any response observed in PLT, HGB, or ANC values, or as any of the following: a doubling of the baseline count, a return to normal counts in one or two cell lines, an increase of 20×10^9^/L in PLT count if baseline count was ≤20×10^9^/L, an increase of 30 g/L in HGB level if baseline level was ≤60 g/L, or an increase of 0.5×10^9^/L in ANC if baseline count was <0.5×10^9^/L [12, 25]. For transfusion-dependent patients, transfusion independence for eight consecutive weeks was also considered a PR [12, 25].

The secondary endpoints of this study were relapse, clonal evolution, and safety predictors of both ORR and CRR at 6 months. Safety was assessed by analyzing the incidence and severity of adverse events and was classified according to the National Cancer Institute Common Toxicity Criteria for Adverse Events version 5.0 [26].

### Statistical analysis

The estimated sample size was calculated based on the ORR reported in historical studies [14, 27]. A sample size of 82 patients (41 per group) was estimated to provide the trial with 80% power (two-sided test) to reject the null hypothesis at a 5% significance level. Data analysis was conducted using SPSS (version 25.0) software. Categorical variables were presented as frequencies, whereas continuous variables were presented as medians (range). Intermediate analyses were performed to determine the statistical power of the therapeutic differences between the ATG + CsA + AVA and CsA + AVA groups. Fisher’s exact test, chi-square test, Student’s t-test, and nonparametric rank-sum test were used to assess the statistical significance of the differences between the two groups. Covariate effects on the response rate were evaluated using binary logistic regression analysis. All statistical tests were two-sided, and statistical significance was set at P < 0.05.

## Results

### Baseline characteristics

A total of 90 patients were screened for eligibility. Among them, three were excluded for not meeting the inclusion criteria and one declined to participate. The remaining 86 patients were enrolled in the study. Forty-three patients were assigned to the ATG + CsA +AVA group, and the remaining 43 were assigned to the CsA + AVA group. Two patients withdrew their informed consent before initiating treatment. A diagram of screening and enrolment details was depicted in Supplementary Figure 1. Ultimately, 84 patients were included in the final analysis. Table 1 summarizes the baseline characteristics of patients. The ATG + CsA + AVA group (n = 42) included 18 men (42.9%) and 24 women (57.1%), with a median age of 65 years (range, 60–78 years). The median duration from diagnosis to treatment was one (range, 0.3–30) month. The median HGB, ANC, and PLT values were 59 (33–82) g/L, 0.46 (0.01–1.49) × 10^9^/L, and 6 (1–34) × 10^9^/L, respectively. Thirty-one patients (73.8%) were diagnosed with SAA and 11 patients (26.2%) were diagnosed with VSAA. The frequencies of PNH clone positivity, chromosomal karyotype abnormalities, and myeloid gene mutations were 26.2% (11/42), 11.9% (5/42), and 31% (13/42), respectively (Table 1). The CsA + AVA group (n = 42) included 21 men (50%) and 21 women (50%), with a median age of 65 years (range, 60–84) years. The median duration from diagnosis to treatment was 1.3 (range, 0.2–35.8) months. The median HGB, ANC, and PLT values were 58 (25–79) g/L, 0.63 (0.04–1.30) × 10^9^/L and 8 (1–43) × 10^9^/L, respectively. Thirty-six patients (85.7%) were diagnosed with SAA, and six (14.3%) were diagnosed with VSAA. The frequencies of PNH clone positivity, chromosomal karyotype abnormalities, and myeloid gene mutations were 14.3% (6/42), 14.3% (6/42), and 23.8% (10/42), respectively. The baseline clinical features of the two groups were comparable (Table 1). The details of the myeloid gene mutation were provided in Supplementary Table 1. The median follow-up duration was 13.3 (range, 0.3–17) months for the ATG + CsA + AVA group, and 13 (range, 4.7–17) months for the CsA + AVA group, respectively (P = 0.872, Table 1).

**Table 1:** Clinical features of patients.

Clinical features	All (*N* = 84)	ATG+CsA+AVA (*n* = 42)	CsA+AVA (*n* = 42)	*P* value
Median age (range), yrs	65 (60–84)	65.5 (60–78)	65 (60–84)	0.381
Male/female (*n*)	39/45	18/24	21/21	0.512
The median duration from diagnosis to treatment (days, range)	1.1 (0.2–35.8)	1 (0.3–30)	1.3 (0.2–35.8)	0.138
Severity classification, no. (%)				0.175
SAA	67 (79.8%)	31 (73.8%)	36 (85.7%)	
VSAA	17 (20.2%)	11 (26.2%)	6 (14.3%)	
HGB (g/L)	59 (25–82)	59 (33–82)	58 (25–79)	0.694
PLT (×10^12^/L)	7.5 (1–43)	6 (1–34)	8 (1–43)	0.119
WBC (×10^9^/L)	2.01 (0.42–4.04)	2.12 (0.42–4.04)	2.01 (0.74–3.67)	0.947
ANC (×10^9^/L)	0.54 (0.01–1.49)	0.46 (0.01–1.49)	0.63 (0.04–1.30)	0.153
Fer (μg/L)	903.5 (11–6409)	713.45 (165–4456.3)	1060.5 (11–6409)	0.195
PNH clone-positive, no. (%)	17 (20.2%)	11 (26.2%)	6 (14.3%)	0.175
PNH clone size (granulocyte flare, %)	6.5 (1.2–18)	3.8 (1.2–17.2)	7.75 (1.4–18)	0.451
Chromosomal karyotype abnormality, no. (%)	11 (13.1%)	5 (11.9%)	6 (14.3%)	0.746
-Y	3 (3.6%)	2 (4.8%)	1 (2.4%)	1.000
del(13)(q12q22)	2 (2.4%)	0 (0.0%)	2 (4.8%)	0.494
+Y	1 (1.2%)	1 (2.4%)	0 (0.0%)	1.000
+8	1 (1.2%)	1 (2.4%)	0 (0.0%)	1.000
+19	1 (1.2%)	0 (0.0%)	1 (2.4%)	1.000
-X	1 (1.2%)	0 (0.0%)	1 (2.4%)	1.000
9qh+	1 (1.2%)	0 (0.0%)	1 (2.4%)	1.000
t(8;11)(q24;p15)	1 (1.2%)	1 (2.4%)	0 (0.0%)	1.000
Myeloid gene mutation, no. (%)	23 (27.4%)	13 (31.0%)	10 (23.8%)	0.463
The median follow-up time (months, range)	13 (0.3–17)	13.3 (0.3–17)	13 (4.7–17)	0.872

*ATG* antithymocyte globulin, *CsA* cyclosporine A, *AVA* avatrombopag, *HGB* hemoglobin, *WBC* white blood cell, *PLT* platelet, *ANC* absolute neutrophil count, *Fer* ferritin, *PNH* paroxysmal nocturnal hemoglobinuria.

### Patient responses

Of the 84 patients enrolled, one patient in the ATG + CsA + AVA group died at 9 days after initiating treatment, and one in the CsA + AVA group died at 141 days. Therefore, a total of 83 patients were included in the efficacy analysis at 3 months, and a total of 82 patients were included at 6 months. Sixty-two patients (31 in each group) completed the entire 12-month treatment period.

**Fig. 1:**
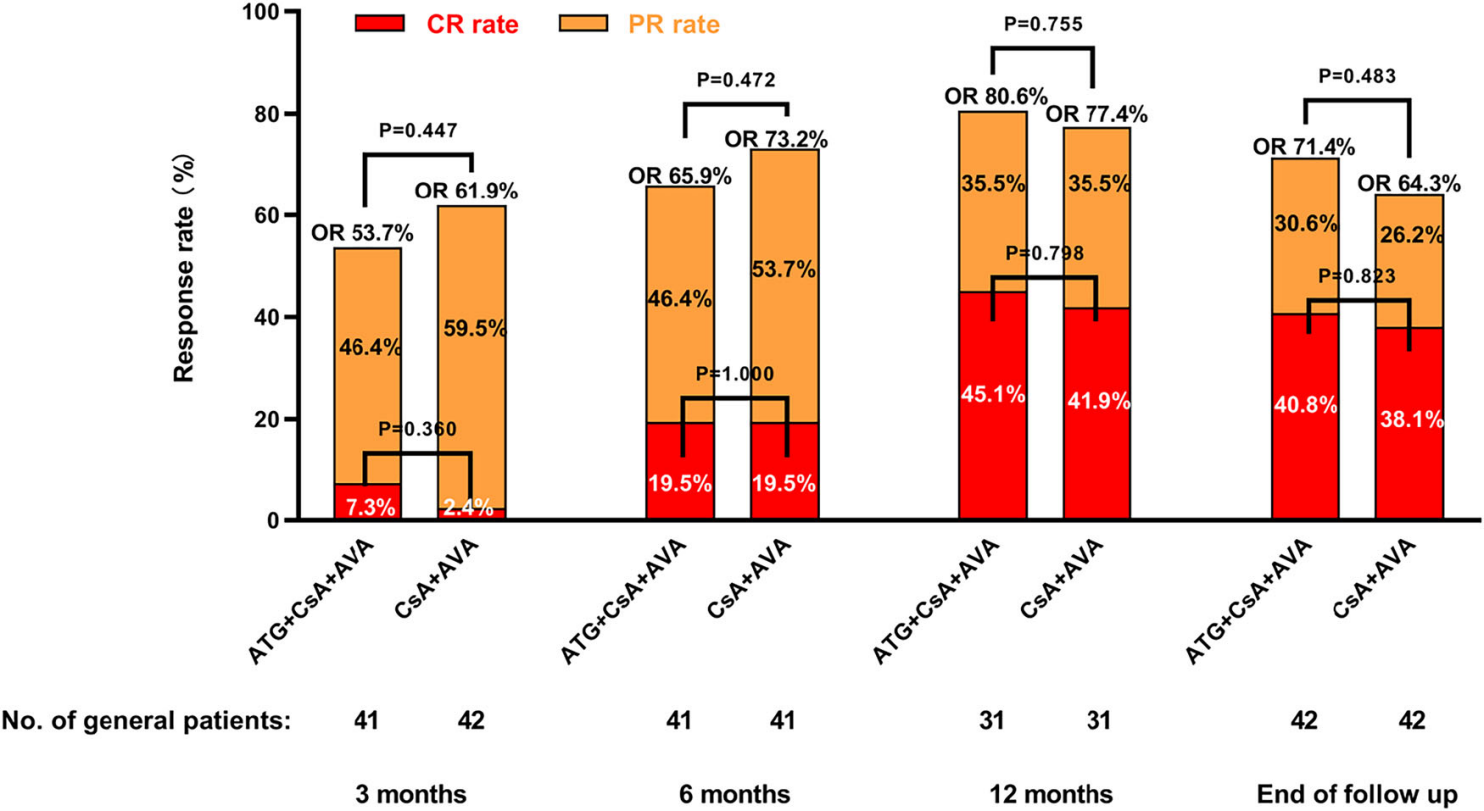
Comparison of efficacy between patients treated with ATG+CsA+AVA and CsA+AVA at different time points. The rates of OR and CR of the two groups were not statistically different at any time point (På 0.05). ATG antithymocyte globulin, CsA cyclosporine A, AVA avatrombopag, CR complete response, PR partial response, OR overall response rate.

The ORR at 3, 6, and 12 months and at the end of follow-up were 53.7%, 65.9%, 80.6%, and 71.4%, respectively, in the ATG + CsA + AVA group, and 61.9%, 73.2%, 77.4%, and 64.3%, respectively, in the CsA + AVA group. The CRR at these time points were 7.3%, 19.5%, 45.1%, and 40.8%, respectively, in the ATG + CsA + AVA group and 2.4%, 19.5%, 41.9%, and 38.1%, respectively, in the CsA + AVA group. The ORR and CRR at 3, 6, and 12 months, and at the end of follow-up were comparable between both groups (P > 0.05, Fig. 1). The median time to objective response (OR) was two (1–7) months in the ATG + CsA + AVA group, and three (1–6) months in the CsA + AVA group (P = 0.522). The median time to CR was 7 (2–13) months in the ATG + CsA + AVA group, and 6.5 (3–12) months in the CsA + AVA group (P = 0.830).

### Predictive factors for ORR and CRR at 6 months

We further analyzed the factors that may influence ORR and CRR at the 6-month mark. We compared factors such as sex, age, duration from diagnosis to treatment, SAA/ VSAA ratio, baseline blood counts, serum ferritin levels, presence of PNH clones, variations in treatment regimens (ATG + CsA + AVA *versus* CsA + AVA), and investigated whether ORR or CRR achieved at 3 months had an impact on achieving OR or CR at 6 months. We identified that most of the above-mentioned characteristics were comparable between the two groups, with the exception of baseline PLT and ANC, and the 3-month ORR, which were significantly higher in patients who achieved OR at 6 months (P = 0.007, 0.030, and 0.000, respectively, Table 2). Similarly, when considering the 3-month ORR and CRR values as predictors for the 6-month CRR, they were found to be higher in patients who achieved CR at 6 months (P = 0.001 and 0.022, respectively, Table 3). However, when factors with P < 0.05 were included in the multivariable regression analysis, no statistically significant factors were observed for the 6-month ORR, however, the 3-month ORR was an independent predictive factor for the 6-month CRR (P = 0.019).

**Table 2:** Factors that may affect the 6-month ORR.

Clinical features	Patients achieved OR (*n* = 57)	Patients not achieved OR (*n* = 25)	*P* value
Median age (range), yrs	64 (60–84)	67 (60–75)	0.266
Male/female (*n*)	28/29	10/15	0.446
The median duration from diagnosis to treatment (days, range)	1.3 (0.2–35.8)	0.7 (0.3–30.7)	0.281
Severity classification, no. (%)			0.707
SAA	47 (82.5%)	19 (76%)	
VSAA	10 (17.5%)	6 (24%)	
HGB (g/L)	59 (33–79)	58 (25–82)	0.503
PLT (×10^12^/L)	9 (1–43)	5 (1–20)	0.007
WBC (×10^9^/L)	2.12 (0.74–4.04)	1.81 (0.42–3.77)	0.197
ANC (×10^9^/L)	0.62 (0.04–1.49)	0.45 (0.04–0.96)	0.030
Fer (μg/L)	921.3 (11–6409)	715 (321–4456.3)	0.691
PNH clone–positive, no. (%)	14 (24.6%)	3 (12%)	0.196
Chromosomal karyotype abnormality, no. (%)	7(12.3%)	4 (16%)	0.918
Myeloid gene mutation, no. (%)	16 (28.1%)	6 (24%)	0.702
Treatment, no. (%)			0.472
ATG+CsA+AVA	27 (47.4%)	14 (56%)	
CsA+AVA	30 (52.6%)	11 (44%)	
3-month response, no. (%)			
ORR	48 (84.2%)	0 (0%)	0.000
CRR	4 (7.0%)	0 (0%)	0.423

*ATG* antithymocyte globulin, *CsA* cyclosporine A, *AVA* avatrombopag, *OR* overall response, *ORR* overall response rate, *CRR* complete response rate, *HGB* hemoglobin, *WBC* white blood cell, *PLT* platelet, *ANC* absolute neutrophil count, *Fer* ferritin, *PNH* paroxysmal nocturnal hemoglobinuria.

**Table 3:** Factors that may affect the 6-month CRR.

Clinical features	Patients achieved CR (*n* = 16)	Patients not achieved CR (*n* = 66)	*P* value
Median age (range), yrs	64.5 (60–82)	66 (60–84)	0.916
Male/female (*n*)	9/7	29/37	0.376
The median duration from diagnosis to treatment (days, range)	0.7 (0.3–11.8)	1.3 (0.2–35.8)	0.461
Severity classification, no. (%)			0.662
SAA	14 (87.5%)	52 (78.8%)	
VSAA	2 (12.5%)	14 (21.2%)	
HGB (g/L)	54 (33–77)	60 (25–82)	0.163
PLT (×10^12^/L)	7 (1–26)	8(1–43)	0.389
WBC (×10^9^/L)	1.79 (0.74–4.04)	2.04 (0.42–3.77)	0.680
ANC (×10^9^/L)	0.56 (0.15–0.94)	0.54 (0.04–1.49)	0.935
Fer (μg/L)	1022.25 (165–2818)	903.5 (11–6409)	0.888
PNH clone-positive, no. (%)	3 (18.8%)	14 (21.2%)	1.000
Chromosomal karyotype abnormality, no. (%)	2(12.5%)	9 (13.6%)	1.000
Myeloid gene mutation, no. (%)	6 (37.5%)	16 (24.2%)	0.448
Treatment, no. (%)			1.000
ATG+CsA+AVA	8 (50%)	33 (50%)	
CsA+AVA	8 (50%)	33 (50%)	
3-month response, no. (%)			
ORR	15 (93.8%)	33 (50%)	0.001
CRR	3 (18.8%)	1 (1.5%)	0.022

*ATG* antithymocyte globulin, *CsA* cyclosporine A, *AVA* avatrombopag, *OR* overall response, *ORR* overall response rate, *CRR* complete response rate, *HGB* hemoglobin, *WBC* white blood cell, *PLT* platelet, *ANC* absolute neutrophil count, *Fer* ferritin, *PNH* paroxysmal nocturnal hemoglobinuria.

### Relapse and clonal evolution

During the 13.3 (range, 0.3–17) and 13 (range, 4.7–17) months (P = 0.872) follow-up duration for the ATG + CsA + AVA and CsA + AVA groups, respectively, the relapse rate was 8.1% (5/62) for the entire cohort. Two of the 32 (6.3%) patients in the ATG + CsA + AVA group relapsed at 9 and 13 months. The patient who relapsed at 9 months, initially had no genetic or chromosomal abnormalities, later developed del(7q) at 13 months and progression to MDS-LB. Three of the 30 (10%) patients in the CsA + AVA group relapsed within a median of 15 months (range, 12–16 months). One patient had a TET2 mutation initially and later acquired an additional ASXL1 mutation at 4 months, and finally progressed to AML. No other clonal evolution was observed. Additionally, no significant differences in the relapse (P = 0.667) or clonal evolution rates (P = 1.000) were observed between the two groups.

### Adverse events and final outcome

Overall, 27 of the 42 patients (64.3%) who received the ATG + CsA + AVA regimen, and 15 of the 42 patients (35.7%) who received the CsA + AVA regimen experienced adverse events (of any grade) during the treatment period (P = 0.009, Table 4). The most frequently reported adverse events (of any grade) in patients treated with ATG + CsA +AVA were infection (26.2%), increased bilirubin levels (16.7%), infusion reactions (16.7%), cardiac toxicity (16.7%), and renal dysfunction (14.3%). The most frequently reported adverse events (of any grade) in patients treated with CsA + AVA were gastrointestinal disorders (14.3%), fatigue (9.5%), headache (9.5%), and renal dysfunction (4.8%). The ATG + CsA + AVA group exhibited significantly higher rates of infection and cardiac toxicity compared to the CsA + AVA group (P = 0.019, P = 0.012, respectively). Eleven (26.2%) patients in the ATG + CsA + AVA group experienced severe adverse events (SAE), including one death due to fungal lung infection and alveolar hemorrhage, four days after the completion of ATG therapy, whereas four (9.5%) patients in the CsA + AVA group experienced SAE (P = 0.047). Most adverse events resolved after dose adjustment or symptomatic treatment.

**Table 4:** Adverse effects treated with ATG+CsA+AVA and CsA+AVA.

Adverse events, *N* (%)	All (*N* = 84)	ATG+CsA+AVA group (*N* = 42)	CsA+AVA group (*N* = 42)	*P* value
No. of patients with AEs	42 (50.0%)	27 (64.3%)	15 (35.7%)	0.009
Grade ≥3	10 (11.9%)	8 (19.0%)	2 (4.8%)	0.043
Grade <3	36 (42.9%)	21 (50.0%)	15 (35.7%)	0.186
No. of patients with SAEs	15 (17.9%)	11 (26.2%)	4 (9.5%)	0.047
Infection	14 (16.7%)	11 (26.2%)	3 (7.1%)	0.019
Gastrointestinal disorders	10 (11.9%)	4 (9.5%)	6 (14.3%)	0.738
Fatigue	9 (10.7%)	5 (11.9%)	4 (9.5%)	1.000
Bilirubin increase	8 (9.5%)	7 (16.7%)	1 (2.4%)	0.057
Renal dysfunction	8 (9.5%)	6 (14.3%)	2 (4.8%)	0.265
Infusion reaction	7 (8.3%)	7 (16.7%)	0 (0%)	0.012
Headache	7 (8.3%)	3 (7.1%)	4 (9.5%)	1.000
Cardiac toxicity	7 (8.3%)	7 (16.7%)	0 (0%)	0.012
Elevated liver enzymes	4 (4.8%)	3 (7.1%)	1 (2.4%)	0.616
Serum sickness	3 (3.6%)	3 (7.1%)	0 (0%)	0.241
Gingival hyperplasia	3 (3.6%)	1 (2.4%)	2 (4.8%)	1.000
Edema	3 (3.6%)	1 (2.4%)	2 (4.8%)	1.000
Ulcer of the oral cavity	3 (3.6%)	2 (4.8%)	1 (2.4%)	1.000

*ATG* antithymocyte globulin, *CsA* cyclosporine A, *AVA* avatrombopag, *AE* adverse events.

Three deaths (7.1% of patients) were reported in the ATG + CsA + AVA group. One patient died of pulmonary infection four days after completing ATG treatment, one died of infection at 10 months; and one died of disease progression at 11 months, due to lack of therapeutic efficacy. Similarly, three deaths (7.1% of patients) were reported in the CsA + AVA group. One patient died of disease progression to AML at 4.7 months, and the other two patients died of concurrent pulmonary infections at 14 and 15 months. No significant differences in deaths were observed between the two groups (P = 1.000). Of note, one case of early mortality was observed on day 9 post-treatment. The patient was diagnosed with VSAA and treated with CsA + ATG + AVA, along with posaconazole prophylaxis for fungal infections. She showed no signs of active infection prior to treatment, but developed a pulmonary infection after completing ATG therapy. Imaging studies and sputum culture (which identified Gram-negative bacilli) confirmed a bacterial pneumonia. The infection rapidly progressed to fulminant sepsis, triggering catastrophic clinical deterioration. Despite prompt antibiotic therapy and supportive care, the patient rapidly developed refractory septic shock and succumbed to irreversible cardiopulmonary failure.

## Discussion

This is the first prospective randomized controlled study comparing the treatment of older adults with SAA using ATG + CsA + AVA and CsA + AVA regimens, in light of the fact that very limited data are available on the use of AVA for newly diagnosed SAA, particularly in older patients [20, 28–30]. Our study emphasized that combining both CsA and AVA achieved comparable efficacy to ATG + CsA + AVA with superior safety for older adults with SAA.

For our primary endpoints, we identified that the ORR and CRR at 6 months were comparable between patients treated with ATG + CsA + AVA and those treated with CsA + AVA, with ORR and CRR values of 65.9% and 19.5%, respectively. Li et al. retrospectively compared the efficacy of the CsA + ATG + AVA regimen in 42 patients with SAA with that of ATG + CsA regimen in 84 patients. They reported that patients treated with CsA + ATG + AVA had higher ORR and CRR values at 3 and 6 months [30]. The 6-month ORR and CRR for patients treated with ATG + CsA + AVA in their study were 71.4% and 31%, respectively [30]. These results were slightly higher than ours, which is probably due to the patient diversity between both studies; our patients were all older adults, whereas the patients in their study were of differing ages. Similarly, in the RACE study, patients in the CsA + ATG + EPAG group achieved a 6-month ORR of 68%, and a CRR of 32%, with the CRR further increasing to 52% at 12 months [13] The slightly higher CRR in the RACE study may be explained by the variations in TPO-RA types, or the variations in age. In the SOAR study, 54 patients newly diagnosed with SAA were treated with CsA + EPAG [31]. Among them, patients aged ≥60 years had an ORR of 45% and a CRR of 9% at 6 months, which were lower than the 73.2% ORR and 19.5% CRR at 6 months of patients treated with CsA + AVA in our study. These differences may be attributed to the variations in type of TPO-RA used, baseline characteristics, or coexisting diseases in patients involved in the two studies, such as the lower proportion of patients with VSAA in our cohort. Several retrospective studies have focused on the use of CsA + TPO-RAs regimens across different age ranges and have shown inconsistent results. For example, a retrospective study of 14 patients with SAA treated with CsA + EPAG with a median follow-up period of 23 months had an ORR of 79% [32]. Zhang et al. reported a comparable ORR of 76.9%, and a higher CRR of 34.6% at 6 months in older adult transfusion-dependent non-severe aplastic anemia (TD-NSAA) patients treated with CsA + AVA [27]. Very few studies have compared the efficacies of CsA + ATG + TPO-RAs and CsA + TPO-RAs. Fattizzo et al. conducted a retrospective study comparing treatment regimens for AA and demonstrated that, among older adults, CsA + EPAG exhibited a superior risk/benefit ratio than other regimens, such as ATG + CsA, ATG + CsA + EPAG, and androgens [33]. Our previous retrospective comparison of ATG + CsA + EPAG and CsA + EPAG regimens in newly diagnosed patients with AA revealed comparable hematological responses between the two regimens in the SAA subgroup [14]. These findings are consistent with our observations. Due to differences in baseline characteristics, types of AA (such as SAA, TD-NSAA and NSAA), types of TPO-RAs, and study type (prospective or retrospective), our study cannot be directly compared with these studies. For other observation time points, no differences in CRR or ORR were found at 3, 12 months or the end of follow-up, either. In conclusion, our study emphasized that treatment with CsA + AVA, a more practical and easier to use oral alternative, had similar efficacy to ATG + CsA + AVA in treating older patients with SAA.

The toxicity data also supported our conclusions. CsA + AVA showed a better tolerance compared to ATG + CsA + AVA regimens. Adverse events occurred in 64.3% of patients treated with ATG + CsA + AVA, with a 26.2% SAE rate, which were higher than those reported in the CsA + AVA group (35.7% and 9.5%, respectively). In a study by Prabahran et al., 54 older adults with SAA received IST with or without EPAG treatment (18 patients received CsA + ATG, 36 patients received CsA + ATG + EPAG, with all patients receiving ATG). They reported a relatively high incidence rate of 38% for SAE within 6 months post-treatment [34]. In the SOAR study with a follow-up period of up to 24 months, the incidence rates of adverse events and SAE in patients treated with CsA + EPAG were 96% and 50%, respectively [31]. Differences in patient characteristics, TPO-RA type, and follow-up duration might have contributed to these findings. In Zhang’s study, 26 older adults (38.5%) with TD-NSAA treated with CsA + AVA experienced adverse events [27], which is similar to our results. Patients who receive ATG frequently develop ATG infusion reactions and serum sickness, as revealed in our study. The incidence of infection and cardiac toxicity in the ATG + CsA + AVA group was significantly higher compared to the CsA + AVA group. Additionally, the increase in bilirubin levels in the ATG + CsA + AVA group was higher than in the CsA + AVA group, approaching statistical significance. The adverse events associated with each regimen were similar to those reported in other studies [18, 34].

In our study, there were no significant differences in the rates of relapse, clonal evolution, or deaths between the ATG + CsA + AVA and CsA + AVA groups. The relapse rate in the ATG group was 6.25%. A study by Townsley et al. revealed that with a median follow-up period of 2 years, the relapse rate was 14% in patients with SAA treated with ATG + CsA + EPAG [12]. The RACE study reported a cumulative relapse rate of 19% at two years for patients treated with CsA + ATG + EPAG [13]. It was also reported that 27% of patients developed new or additional mutations, and 8.3% of patients died during the 2-year period [13]. However, Li et al. reported that for patients with SAA treated with CsA + ATG + AVA, during a median follow-up period of 14 months, only one among 42 patients relapsed, with no clonal evolutions or deaths [30]. In Zhang’s study, the relapse rate was 10%, whereas the mortality rate was 5%, at a median follow-up period of 10 months [27]. According to most published studies, the general clonal evolution rate in patients with SAA was 10–15%, with myeloid malignant transformation typically occurs approximately 5 years after diagnosis [35–37]. In our study, two clonal evolutions were observed. One patient subsequently developed del(7q) with progression to MDS-LB, while the other acquired an additional ASXL1 mutation, ultimately progressing to AML. They both had poor responses to the treatments, similar to the reported clonal evolution patterns in SAA [37].

We also investigated predictive factors potentially affecting the response rates. We identified that although the values of PLT, ANC, and 3-month ORR were significantly higher in patients who achieved OR at 6 months, the multivariate analysis did not reveal any statistically significant factors. However, the 3-month ORR was the only predictive factor for the 6-month CRR. Li et al. reported that, regarding patients receiving CsA + ATG + AVA, a longer interval between disease onset (cytopenia) and ATG treatment (Dis–ATG) is a predictor of a poorer hematologic response at 6 months in patients with SAA [30]. Predictive factors varied across different studies; residual hemopoiesis, time to treatment, and early reactions are frequent factors reported in the literature [31, 38].

Our study had some limitations. Only two centers were involved in the study due to the limited number of patients with SAA in China; however, our center and the Institute of Hematology and Blood Diseases Hospital are the two leading hospitals providing care for this disease in China. The relatively short follow-up duration precluded the assessment of long-term outcomes, including overall survival and the potential of clonal evolution. However, these findings encourage further large-scale prospective studies with longer follow-up periods.

To summarize, our study showed that both ATG + CsA + AVA and CsA + AVA regimens are safe and effective as the first-line treatments for older patients newly diagnosed with SAA. CsA + AVA not only achieved comparable efficacy to ATG + CsA + AVA, but also demonstrated better tolerability. Given these findings, the CsA + AVA regimen shows significant potential as a first-line treatment for newly diagnosed older adults with SAA.

## Supplementary information

supplementary information

## Data Availability

All the data are available in the main text, and all the detailed metadata are available upon reasonable request from the corresponding author.
