# Supplementary material for: Efficacy and safety of immunosuppressive therapy versus cyclosporine combined with avatrombopag in older adults with severe aplastic anemia: a multicenter prospective study
Source: Blood Cancer J. 2025 Jul 5;15(1):119. doi: 10.1038/s41408-025-01328-3 (PMC12228700; doi:10.1038/s41408-025-01328-3)
Supplement: Supplementary file 1 — supplementary information [file 41408_2025_1328_MOESM1_ESM.docx]

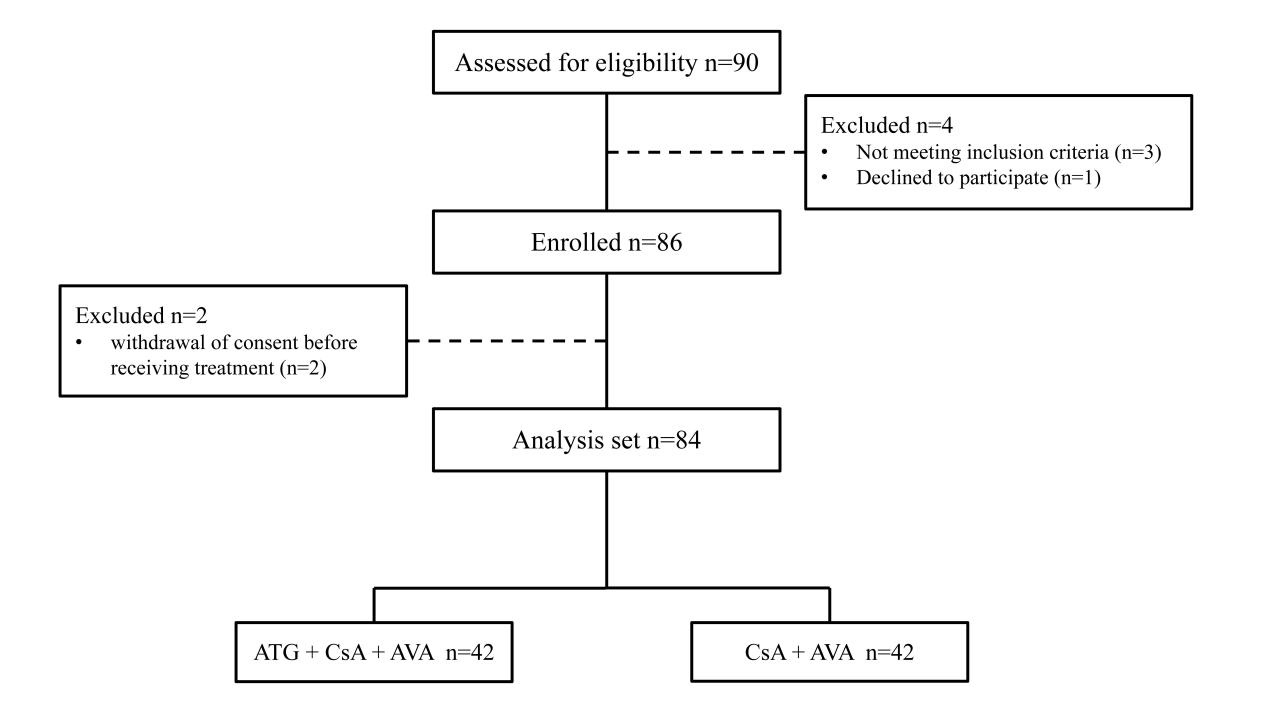


**Supplementary Figure 1** CONSORT diagram illustrating patient screening.

**Supplementary Table 1.** Details of the Myeloid gene mutation

| Myeloid gene mutation | All(N=84) | |  | ATG+CsA+AVA (n=42) | |  | CsA+AVA (n=42) | |  | P value ^#^ |
| --- | --- | --- | --- | --- | --- | --- | --- | --- | --- | --- |
|  | No. (%) ^*^ | Median VAF (%, range) |  | No. (%) | Median VAF (%, range) |  | No. (%) | Median VAF (%, range) |  |  |
| PIGA | 10 (11.9%) | 5.4 (1.2-13.4) |  | 6 (14.3%) | 5.2 (1.2-12.9) |  | 4 (9.5%) | 7.9 (3.5-13.4) |  | 0.500 |
| ASXL1 | 7 (8.3%) | 9.1 (0.9-11.3) |  | 5 (11.9%) | 9.4 (3.1-11.3) |  | 2 (4.8%) | 5.0 (0.9-9.1) |  | 0.433 |
| BCOR/BCORL1 | 5 (6.0%) | 11.2 (2.9-15.5) |  | 2 (4.8%) | 10.1 (3.3-16.8) |  | 3 (7.1%) | 11.2 (2.9-15.5) |  | 1.000 |
| DNMT3A | 5 (6.0%) | 8.7 (1.8-12.7) |  | 3 (7.1%) | 8.7 (7.4-12.7) |  | 2 (4.8%) | 5.4 (1.8-8.9) |  | 1.000 |
| TET2 | 4 (4.8%) | 7.2 (2-10.1) |  | 1 (2.4%) | 6.5 |  | 3 (7.1%) | 7.8 (2-10.1) |  | 0.616 |
| TP53 | 2 (2.4%) | 9.6 (8.2-11.0) |  | 1 (2.4%) | 11.0 |  | 1 (2.4%) | 8.2 |  | 1.000 |
| EZH2 | 1 (1.2%) | 1.2 |  | 1 (2.4%) | 1.2 |  | 0 | - |  | 1.000 |
| U2AF1 | 1 (1.2%) | 5.5 |  | 1 (2.4%) | 5.5 |  | 0 | - |  | 1.000 |
| RUNX1 | 1 (1.2%) | 7.0 |  | 0 | - |  | 1 (2.4%) | 7.0 |  | 1.000 |
| SRSF2 | 1 (1.2%) | 13.1 |  | 0 | - |  | 1 (2.4%) | 13.1 |  | 1.000 |

ATG: antithymocyte globulin; CsA: cyclosporine A; AVA: avatrombopag

VAF: variant allele frequency

*The number of individuals with gene mutations (the percentage of individuals with gene mutations among the total number of individuals)

#P: <0.05 indicates a statistically significant difference in gene mutation rates between the ATG+CsA+AVA and CsA+AVA groups
